# Integrated analysis of MYH6, MYH7, and MYH7B expression and their associated microRNAs in human end-stage heart failure

**DOI:** 10.3389/fcvm.2026.1879458

**Published:** 2026-08-07

**Authors:** Zdenko Červenák, Filip Červenák, Simona Valášková, Nikola Chomaničová, Nikoleta Katreničová, Michal Hulman, Andrea Gažová, Jan Kyselovic

**Affiliations:** 1 5th Department of Internal Medicine, Faculty of Medicine, Comenius University, Bratislava, Slovakia; 2 Department of Genetics, Faculty of Natural Sciences, Comenius University, Bratislava, Slovakia; 3 Institute of Pharmacology and Clinical Pharmacology, Faculty of Medicine, Comenius University, Bratislava, Slovakia; 4 Department of Pharmacology and Toxicology, Faculty of Pharmacy, Comenius University, Bratislava, Slovakia; 5 Department of Cardiac Surgery, Faculty of Medicine, Comenius University and National Institute of Cardiovascular Diseases (NÚSCH), Bratislava, Slovakia

**Keywords:** end-stage heart failure, *MHRT*, *MYH7*, *MYH7b*, myomiR, myosin heavy chain, *SOX6*, transcript ratio

## Abstract

**Background:**

Cardiac contractility is regulated by two myosin heavy chain (MHC) protein isoforms, *α* and β, encoded by the *MYH6* and *MYH7* genes. The intronic regions of these genes encode the microRNAs *miR-208a* and *miR-208b*, which are key regulators of cardiac hypertrophy. Functionally relevant long non-coding RNAs (lncRNAs) have also been identified at these loci, such as those transcribed in the antisense direction from the *MYH7* gene, including the primary *miR-208b* transcript (*MHRT*) originating from an internal *MYH7* promoter. Additionally, *MYH7B*, another cardiac sarcomeric myosin gene, serves as a precursor for *miR-499a-5p*.

**Aim:**

This study aimed to comprehensively analyse the expression profiles of *MYH6, MYH7,* and *MYH7B* genes, microRNAs (*miR-208a-3p, miR-208b-3p, and miR-499a-5p*), and the *lncRNA MHRT* in non-failing and end-stage failing human left ventricles.

**Subjects and methods:**

The relative expression of these transcripts was measured using RT-qPCR in left ventricular samples from 5 non-failing controls and 24 patients with end-stage heart failure, including hypertrophic cardiomyopathy (HCM, *n* = 4), dilated cardiomyopathy (DCM, *n* = 10), and coronary artery disease (CAD, *n* = 10). The findings were validated by comparison with two independent RNA-seq datasets (GSE141910 and GSE116250) for *MYH6*, *MYH7*, *MYH7B*, and *MHRT*.

**Results:**

Statistical analysis of independent RNA-seq datasets confirmed strong correlations among *MYH7, MYH7B,* and *MHRT* transcripts (*p* ≤ 0.0001), a trend also observed in our RT-qPCR cohort. A profound imbalance in the *MYH7B/miR-499-5p* ratio was identified, shifting from ∼250:1 in non-failing controls to ∼12–18:1 in failing hearts. The *miR-208a-3p/miR-208b-3p* expression ratio was heavily skewed toward *miR-208b-3p* (∼50:1) in all groups, regardless of disease status.

**Conclusion:**

Our results suggest that *MYH7, MYH7B*, and *MHRT* transcripts exhibit coordinated expression patterns in human left ventricles, independent of pathological status. We further observed a dissociation between *miR-208a-3p* and its host gene (*MYH6*) under physiological conditions and confirmed altered *miR-499a-5p/MYH7B* relationships, consistent with findings in murine models. Moreover, the expression of two regulatory genes, *SOX6* and *TARBP2*, known to modulate *MYH7* and *MYH7B* in mice, showed that *SOX6* is upregulated in the left ventricles of all pathological groups.

## 1 Introduction

The heart acts as a central hemodynamic pump, ensuring the continuous delivery of oxygen and nutrients to meet the metabolic demands of peripheral tissues. Acute and chronic cardiac insults trigger a spectrum of structural and functional alterations, collectively defined as cardiac remodelling (1). This multi-faceted process encompasses molecular, cellular, and biomechanical adaptations that initially maintain cardiac output under increased workload but eventually transition into maladaptive changes that drive disease progression (2).

A hallmark of cardiac remodelling is cardiomyocyte hypertrophy, an initial adaptive response to sustained hemodynamic stress. However, persistent pathological stimuli—such as hypertension or myocardial infarction—often trigger a transition to maladaptive remodelling, characterised by ventricular dilation, interstitial fibrosis, and impaired contractile efficiency, ultimately leading to heart failure, arrhythmias, or sudden cardiac death (2). These pathological alterations are orchestrated by extensive shifts in the cardiac transcriptome, resulting in increased protein synthesis, a metabolic switch from oxidative phosphorylation toward glycolysis, cytoskeletal reorganisation, and disrupted cardiomyocyte survival and contractility (3–7). Cardiac contractility is primarily regulated by two myosin heavy chain (MHC) protein isoforms, *α*-MHC and β-MHC, encoded by the *MYH6* and *MYH7* genes, respectively (8). Although the *MYH6* and *MYH7* genes are arranged in a head-to-tail tandem configuration and their respective proteins share approximately 93% sequence identity in the motor domain (9), they exhibit distinct functional properties. The *α*-MHC isoform exhibits nearly threefold higher ATPase activity and generates greater power output than β-MHC, supporting slower, more energy-efficient contractions (10–12). Numerous studies have demonstrated that pathological cardiac hypertrophy is associated with the downregulation of *MYH6* and the upregulation of *MYH7*, reflecting disease-associated shifts in MHC isoform ratios (13–15). Clinical interventions, such as beta-blocker therapy in patients with dilated cardiomyopathy (DCM), have been shown to reverse this ratio, where increased MYH6 and decreased MYH7 expression correlate with improved left ventricular ejection fraction (16).

Beyond their roles as structural proteins, both *MYH6* and *MYH7* genes give rise to additional functionally relevant non-coding transcripts. The intronic regions of these genes encode the microRNAs *miR-208a* and *miR-208b*, which are established as key regulators of cardiac hypertrophy (17, 18). Interestingly, a long non-coding RNA (lncRNA) named *MHRT* (Myosin Heavy Chain Associated RNA Transcript) is transcribed in the antisense direction from the *MYH7* locus (19, 20); this transcript also serves as the primary *miR-208b* transcript (*pri-miR-208b*), originating from an internal promoter within the *MYH7* gene (21, 22). Additionally, *MYH7B*, another sarcomeric myosin gene expressed in the myocardium, serves as the host gene for *miR-499a-5p*, although its precise cardiac function remains to be fully elucidated (18).

These transcripts interact within a complex regulatory network that governs cardiac gene expression; specifically, the three MyomiRs derived from these loci—*miR-208a-3p, miR-208b-3p*, and *miR-499a-5p*—have been shown to modulate the *MYH6/MYH7* expression balance under stress conditions in murine models (18). Importantly, *miR-208a-3p* overexpression can induce *MYH7* expression, particularly under hypothyroid conditions (17), whereas its knockout leads to significant *downregulation of MYH7* (18, 23). The lncRNA *MHRT* exerts anti-hypertrophic effects by enhancing *MYH6* expression (20, 24) and modulating myocardin acetylation (25). Furthermore, *MHRT* has been shown to regulate primary *miR-208b-3p* expression in a sex-specific manner (26), while *miR-208b-3p* itself influences *MYH6* and *MHRT* levels in the murine heart (22). Additionally, MYH7B-derived lncRNAs have been proposed to modulate MYH7 gene expression in human induced pluripotent stem cell-derived cardiomyocytes (hiPSC-CMs) (27).

It is important to note that most of these findings originate from rodent models, and the direct applicability of these results to human cardiac physiology may be limited. Notably, profound species-specific differences exist in cardiac MHC isoform expression: adult human left ventricles predominantly express *MYH7*, whereas *Myh6* is the dominant isoform in the ventricles of adult mice and rats (7). Therefore, a detailed investigation of the transcriptional and post-transcriptional regulation of these loci in the human heart is essential to understand their specific roles in human cardiac physiology and pathophysiology.

In this study, we comprehensively analysed the expression profiles of the MYH6, MYH7, and MYH7B genes, their associated microRNAs (miR-208a-3p, miR-208b-3p, and miR-499a-5p), and the lncRNA *MHRT* in non-failing and end-stage failing human left ventricles affected by various cardiomyopathies. Additionally, we assessed the transcriptional profiles of TARBP2 and SOX6, two regulatory genes implicated in modulating myosin heavy chain expression.

## 2 Materials and methods

### 2.1 Tissue samples

Left ventricular (LV) myocardial tissue samples were obtained from failing human hearts (*n* = 24) and non-failing controls (NF, *n* = 5). Failing heart tissues were collected from patients undergoing orthotopic heart transplantation due to end-stage heart failure; their detailed clinical characteristics have been previously reported (28). Samples were classified into three groups based on the underlying aetiology: hypertrophic cardiomyopathy (HCM, *n* = 4; 2 females, 2 males), dilated cardiomyopathy (DCM, *n* = 10; all males), and coronary artery disease (CAD, *n* = 10; all males). All myocardial biopsies were immediately snap-frozen in liquid nitrogen and stored at −80 °C until total RNA extraction.

### 2.2 RNA isolation and cDNA synthesis

Total RNA isolation, assessment of RNA concentration and integrity, cDNA synthesis for mRNA, RT-qPCR conditions, and the selection of reference genes for normalisation were performed as previously described (28). Specific TaqMan Gene Expression Assays used for mRNA quantification are provided in Supplementary SM1 Table 1. The specialised procedures for miRNA expression analysis are detailed in the following section.

### 2.3 Reverse transcription and RT-qPCR for miRNAs

For miRNA analysis, 60 ng of total RNA was reverse transcribed in a total volume of 20 μL using the miRCURY LNA RT Kit (Qiagen GmbH, Germany) according to the manufacturer's instructions. The resulting cDNA was diluted to a final volume of 60 μL. Each sample was amplified in triplicate on a QuantStudio 5 Real-Time PCR System (Thermo Fisher Scientific, USA). Each reaction contained 1 ng of cDNA, the miRCURY LNA SYBR Green PCR Kit, and miRCURY LNA miRNA PCR Assays specific for each target miRNA (Qiagen GmbH, Germany; Supplementary SM1 Table 1). PCR cycling conditions were performed following the manufacturer's protocols.

### 2.4 Normalisation strategy and relative expression analysis

Raw fluorescence data (Rn, normalised to ROX) were processed with LinRegPCR (29) to determine individual amplification efficiencies and Cq values. These were subsequently used to calculate N₀ values (starting concentrations) according to the formula:

$$N_{0}=\mathrm{threshold}/(\mathrm{Efficienc}y^{\mathrm{cq}})$$

For both mRNA and miRNA targets, normalised expression values were calculated as the ratio of the N₀ of the gene of interest to a normalisation factor (NF), using the formula:

$$\begin{array}{rl} & \mathrm{Normalized}\;\mathrm{expression}=\\ & N_{0}(\mathrm{gene}\;\mathrm{of}\;\mathrm{interest})/N_{0}(\mathrm{normalization}\;\mathrm{factor}) \end{array}$$

The NF for mRNA analysis was calculated as the geometric mean of N₀ values for *IPO8*, *POLR2A*, and *PGK1* (28). For miRNA normalisation, the geometric mean of N₀ values for *miR-125b-5p* and *miR-454-3p* (selected for this study) was used. Additionally, N₀ values were used to directly calculate the expression ratios of miRNAs to their corresponding host gene transcripts (*miR-208a-3p/MYH6, miR-208b-3p/MYH7,* and *miR-499a-5p/MYH7B*), as well as the *miR-208a-3p/pre-miR-208a* and *MYH6/pre-miR-208a* ratios across all sample groups.

### 2.5 RNA-seq data retrieval

Normalised RNA-Seq read counts for *MYH6, MYH7, MYH7B*, and the lncRNA *MHRT* in both non-failing and failing human left ventricles were extracted from the publicly available datasets GSE141910 (30) and GSE116250 (31). Data extraction was performed using the GEO2R interactive web tool (32). The extracted expression values and detailed group comparisons are provided in Supplementary SM1 Tables 2–5.

### 2.6 *In-silico* RNA-RNA interaction analysis

Potential intermolecular interactions between *miR-499a-5p* and MYH7B transcripts were analysed using the IntaRNA tool (version 2.0) from the Freiburg RNA Tools platform (33). This analysis was performed to identify putative binding sites and assess the hybridisation energy between the miRNA and its host gene transcript.

### 2.7 Statistical analysis

Data are presented as mean ± SEM. Differences among more than two groups were assessed using the non-parametric Kruskal–Wallis test followed by Dunn's *post hoc* test, while comparisons between two groups were performed using the non-parametric Mann–Whitney *U* test. Correlations between variables were assessed using the Spearman correlation coefficient.

Transcript ratios were calculated in two ways:

1. For mRNA–mRNA and miRNA–miRNA comparisons: unnormalised expression values were used, with results also presented as relative percentages for clarity.
2. For mRNA–miRNA comparisons, ratios were calculated using unnormalised N₀ values.

### 2.8 Ethical statement

This study was conducted in accordance with the principles of the Declaration of Helsinki and was approved by the Ethics Committee of the National Institute for Cardiovascular Diseases, Bratislava, Slovakia (Approval Nos. IK-NP-0021–24/1426/14 and NUSCH EK 126/180509). All patients provided written informed consent after receiving a detailed explanation of the study's purpose and the nature of the experiments. Personal data were handled in strict accordance with the General Data Protection Regulation (GDPR) and the Slovak Republic's legal framework (Personal Data Protection Act No. 18/2018 Coll.).

## 3 Results

### 3.1 Co-regulation of *MYH7*, *MYH7B* and *MHRT* transcription in the left ventricle of the human heart

**Figure 1:**
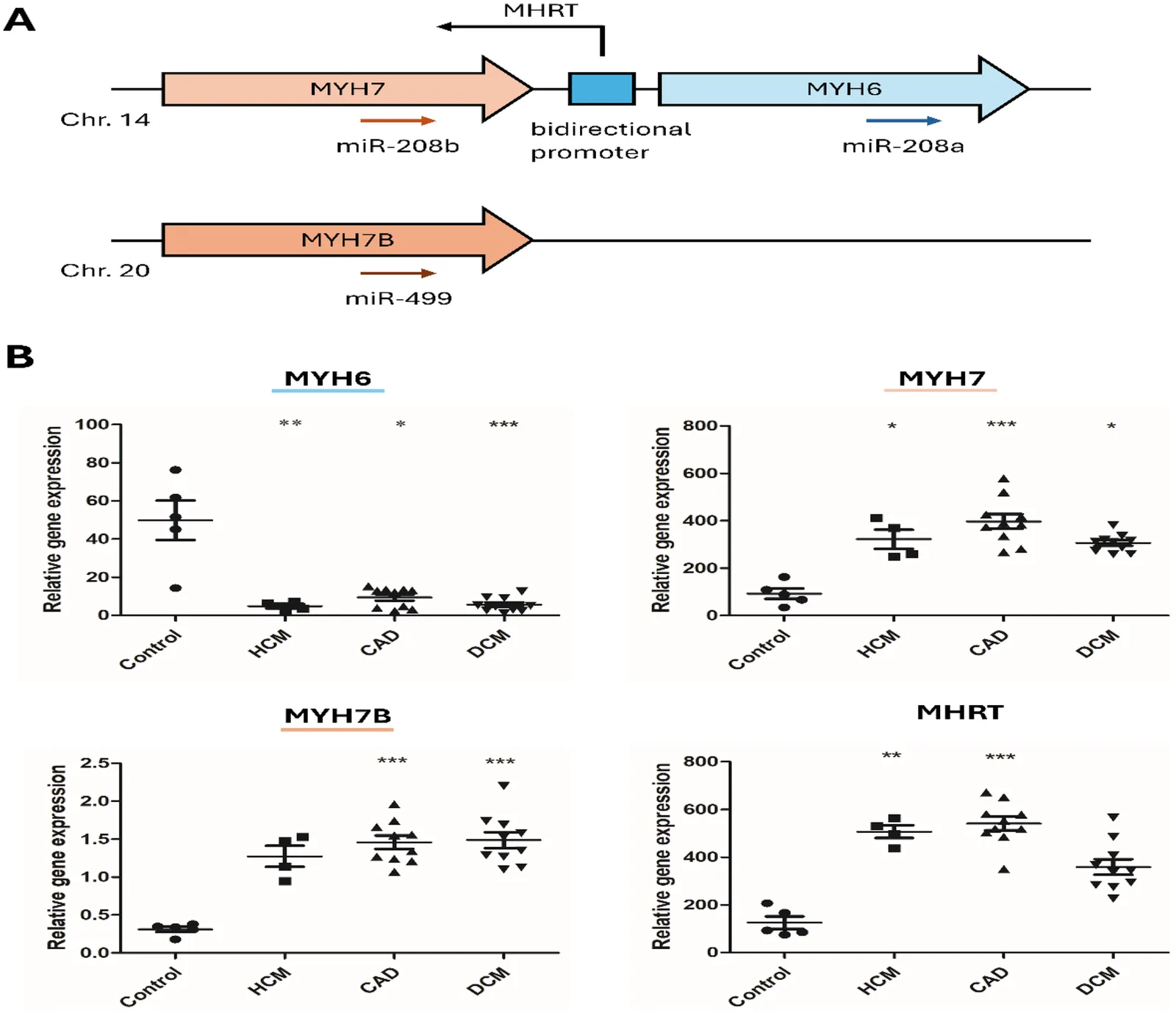
Relative expression of MYH6, MYH7, MYH7B, and *MHRT* transcripts in non-failing and failing human left ventricles. **(A)** Schematic representation of the genomic organisation of the indicated myosin genes and their respective transcripts. **(B)** Plots represent the mean expression ± SEM. Statistical significance is indicated as: ***p < 0.001, **p < 0.01, *p < 0.05. HCM, hypertrophic cardiomyopathy; CAD, coronary artery disease; DCM, dilated cardiomyopathy.

To investigate the complex transcriptional and post-transcriptional regulation of myosin paralogs in the left ventricle of failing human hearts, we first performed RT-qPCR analysis of the *MYH6, MYH7*, and *MYH7B* transcripts, along with the long non-coding RNA *MHRT* (Figures 1A,B).

As expected, *MYH6* expression was consistently reduced across all examined cardiomyopathies, including coronary artery disease (CAD), dilated cardiomyopathy (DCM), and hypertrophic cardiomyopathy (HCM). In contrast, *MYH7, MYH7B*, and *MHRT* were significantly upregulated in the majority of diseased tissues compared to non-failing controls (Figure 1B; Supplementary SM2 Tables 1–4). Notably, *MYH6* expression in healthy controls exhibited a substantially higher standard deviation compared to other myosin transcripts, suggesting greater physiological variability of *MYH6* expression in non-diseased hearts. This observation is consistent with previously published datasets (Supplementary SM3 Table 1) and likely reflects the dynamic, stimulus-responsive nature of *MYH6* transcription. Given the similar expression profiles of *MYH7, MYH7B*, and *MHRT*, we next assessed potential correlations between their transcript levels; however, no statistically significant correlations were observed within individual disease groups, likely due to the limited sample size (Supplementary SM3 Table 2).

**Figure 2:**
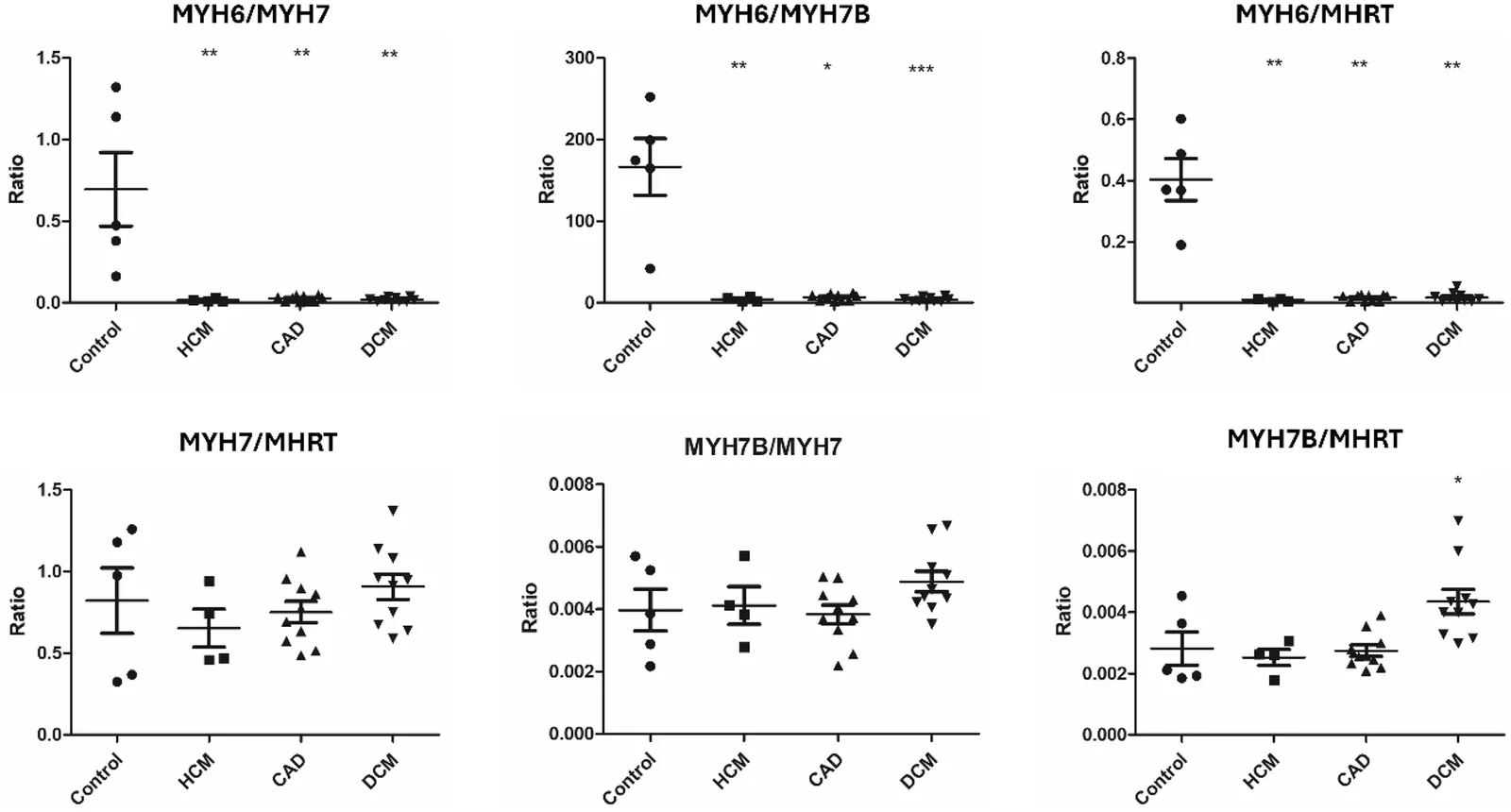
Ratios of normalised relative expression for MYH6, MYH7, MYH7B, and *MHRT* transcripts. Plots represent the mean ratio ± SEM. Statistical significance is indicated as: ***p < 0.001, **p < 0.01, *p < 0.05. HCM, hypertrophic cardiomyopathy; CAD, coronary artery disease; DCM, dilated cardiomyopathy.

Considering the established clinical relevance of the *MYH6/MYH7* ratio in characterising myocardial properties, we further analysed this and additional transcript ratios across all groups (Figure 2). The *MYH6/MYH7, MYH6/MYH7B*, and *MYH6/MHRT* ratios were significantly reduced in all cardiomyopathy groups compared to non-failing controls (Supplementary SM4 Tables 1–3).

Conversely, ratios such as *MYH7/MHRT*, *MYH7B/MYH7*, and *MYH7B/MHRT* remained largely unchanged across groups, except for a minor difference in the *MYH7B/MHRT* ratio within the DCM cohort (Supplementary SM4 Tables 4–6). These results indicate a highly coordinated expression of the *MYH7, MYH7B*, and *MHRT* genes, which is maintained regardless of the underlying disease aetiology.

**Figure 3:**
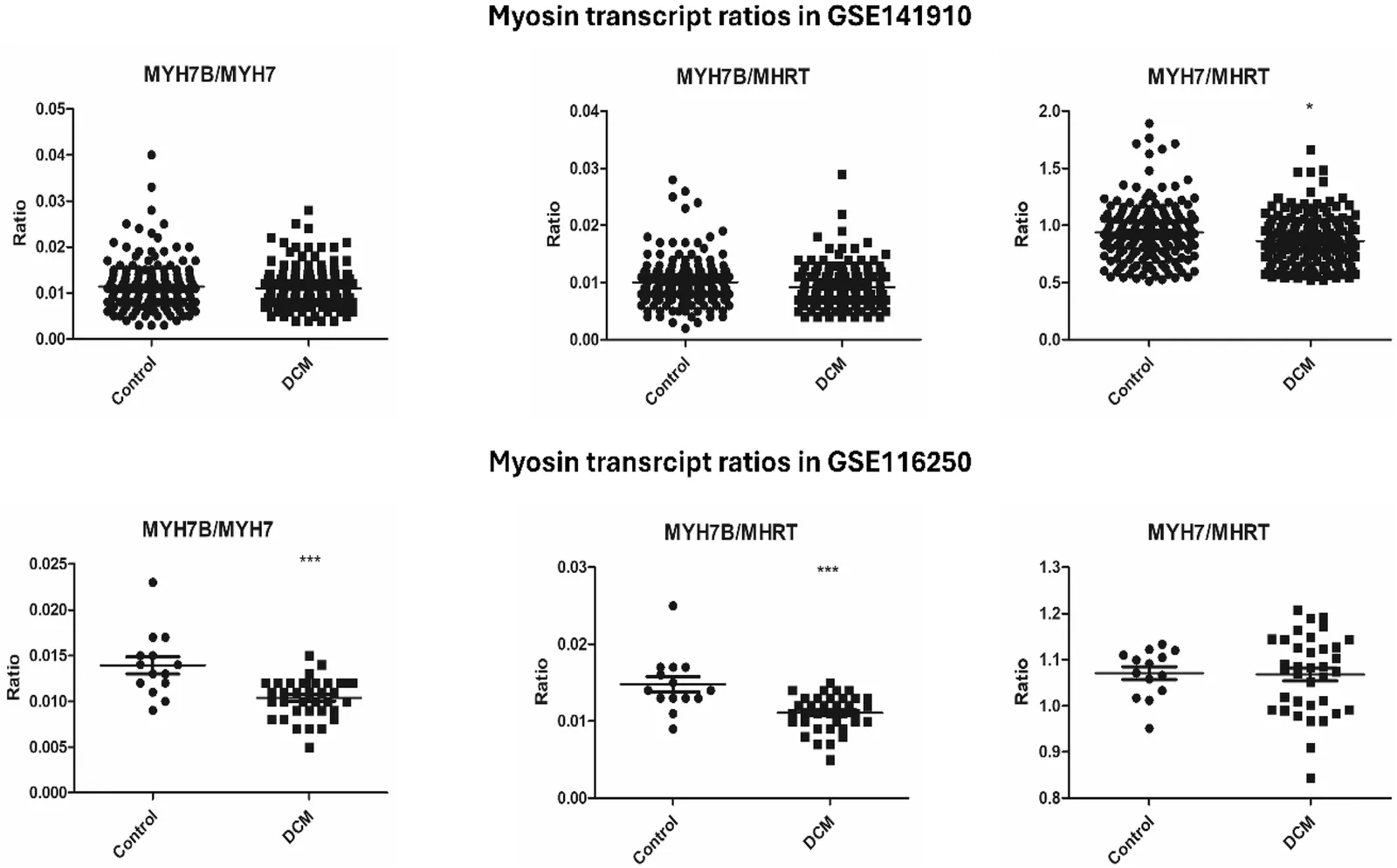
Validation of transcript ratios using independent RNA-Seq datasets (GSE141910 and GSE116250). Data represent normalised expression ratios in non-failing controls and patients with dilated cardiomyopathy (DCM). Statistical significance was determined using the Mann–Whitney test (***p < 0.001, **p < 0.01, *p < 0.05).

To validate these findings, we reanalysed two publicly available RNA-seq datasets, GSE141910 and GSE116250, focusing on non-failing control and DCM samples (30, 31). Correlation analyses in both datasets confirmed strong positive associations between *MYH7*, *MYH7B*, and *MHRT* transcript levels (Supplementary SM5 Table 1), supporting the hypothesis of shared regulatory mechanisms. However, transcript ratio analyses revealed some dataset-specific differences: a significant alteration of the *MYH7/MHRT* ratio in GSE141910, and changes in the *MYH7B/MHRT* and *MYH7B/MYH7* ratios in GSE116250 (Figure 3; Supplementary SM5 Table 2). These observations suggest that while the co-regulation of these genes is evident across different studies, the precise stoichiometry and consistency of this regulation may vary between patient cohorts.

### 3.2 Long non-coding RNA *MHRT* is upregulated in failing human hearts

**Figure 4:**
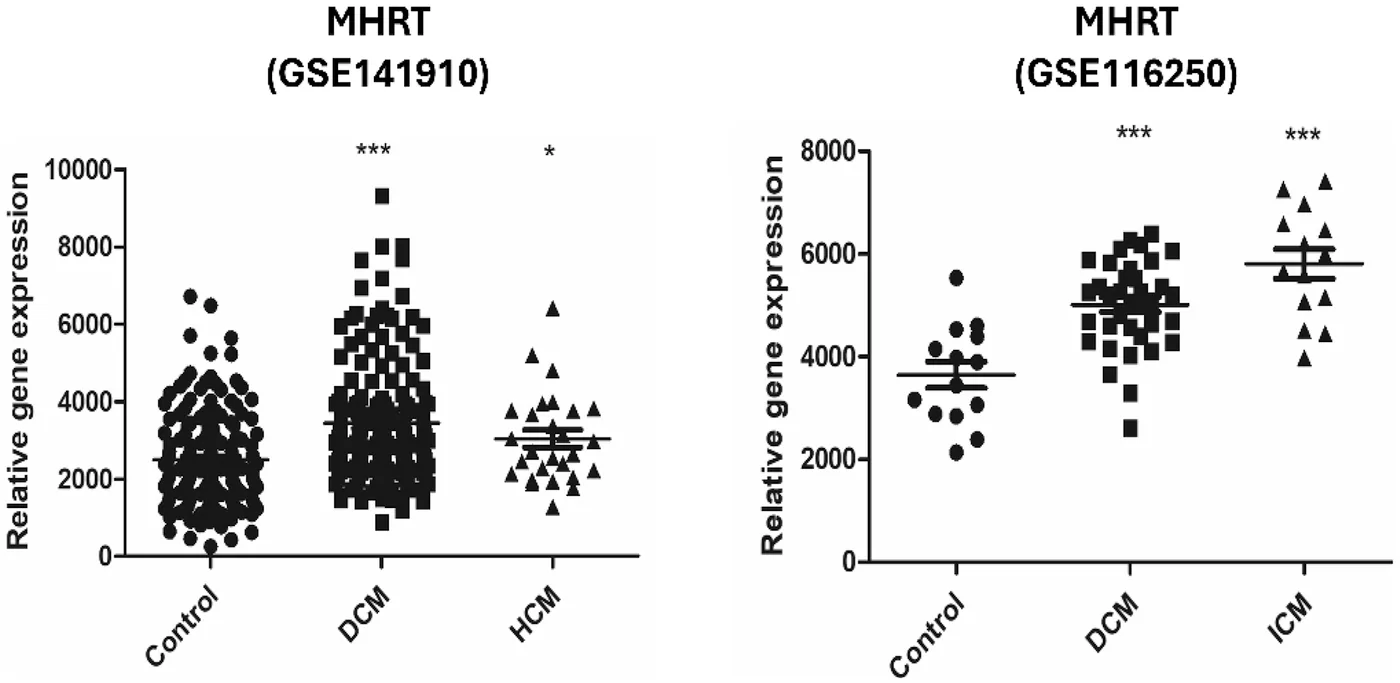
Relative expression of *MHRT* in non-failing and failing human left ventricles across independent datasets. Plots indicate the mean gene expression ± SEM. Statistical significance is indicated as: ***p < 0.001, **p < 0.01, *p < 0.05. HCM, hypertrophic cardiomyopathy; ICM, ischemic cardiomyopathy; DCM, dilated cardiomyopathy.

Our analyses consistently demonstrated that *MHRT* expression is significantly elevated in diseased hearts, including HCM, CAD, and DCM cohorts (Figure 1B; Supplementary SM2 Table 4). This is in line with data from the two aforementioned RNA-seq datasets, which also showed significant upregulation in tissues from patients with ischemic cardiomyopathy (ICM, GSE116250) and HCM (GSE141910) (Figure 4; Supplementary SM5 Table 3). These findings support a potential role for *MHRT* in molecular adaptations underlying cardiac remodelling and hypertrophy across diverse heart diseases.

### 3.3 Expression of *MYH*-derived microRNAs is not coordinately regulated in diseased hearts

**Figure 5:**
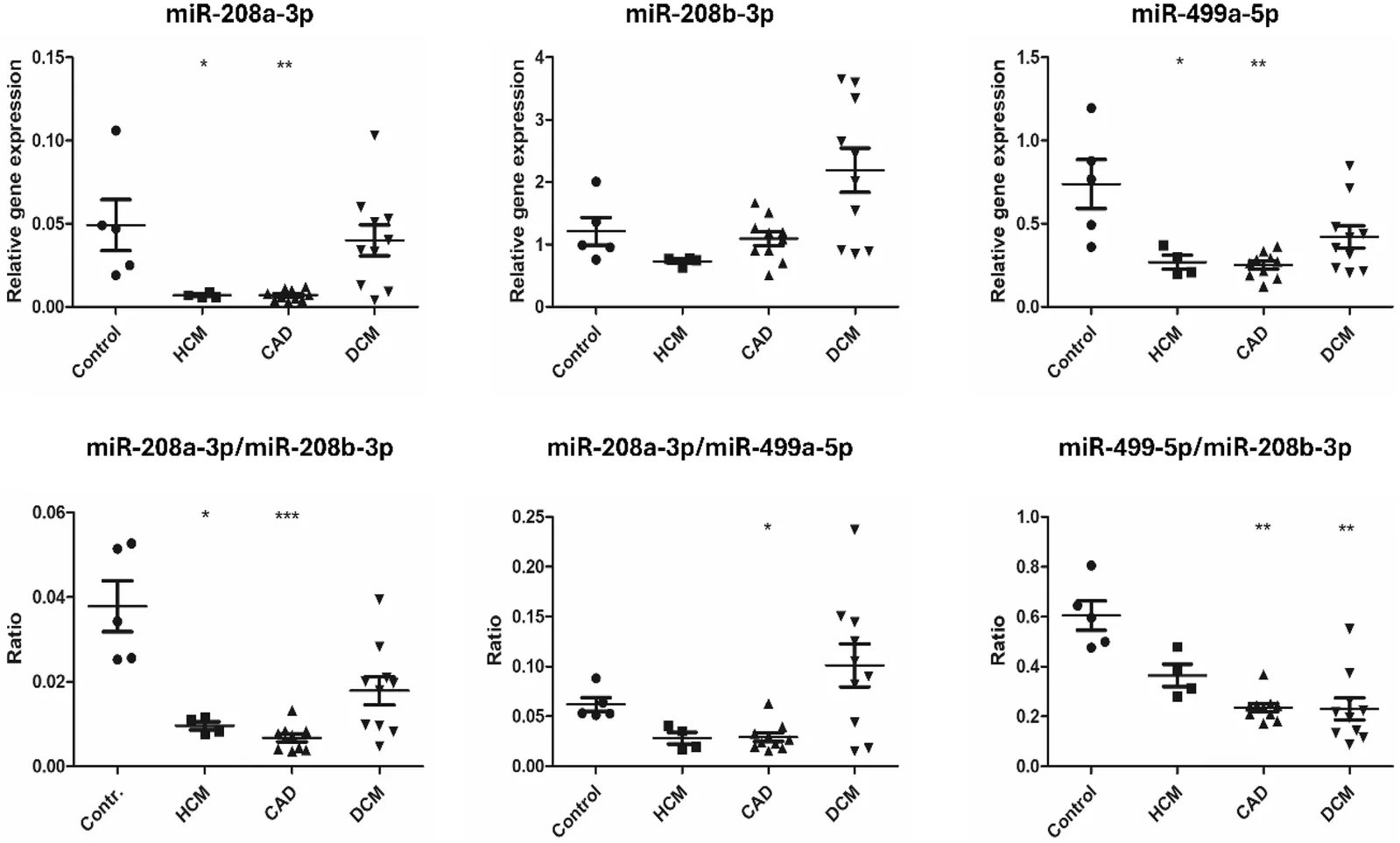
Relative expression of MYH-derived miRNAs and their specific ratios. Plots show normalised expression levels of the indicated miRNAs and their ratios (mean plus/minus SEM). Statistical significance: ***p < 0.001, **p < 0.01, *p < 0.05. HCM, hypertrophic cardiomyopathy; CAD, coronary artery disease; DCM, dilated cardiomyopathy.

To further explore the regulatory landscape of myosin transcripts, we examined the expression of their intronic microRNAs: *miR-208a-3p (MYH6), miR-208b-3p (MYH7),* and *miR-499a-5p (MYH7B)*. Consistent with MYH6 transcript levels, miR-208a-3p was downregulated in diseased tissues, despite its already low basal expression in healthy hearts. A similar downregulation was observed for miR-499a-5p, suggesting comparable regulatory mechanisms (Figure 5; Supplementary SM6 Tables 1–3).

**Figure 6:**
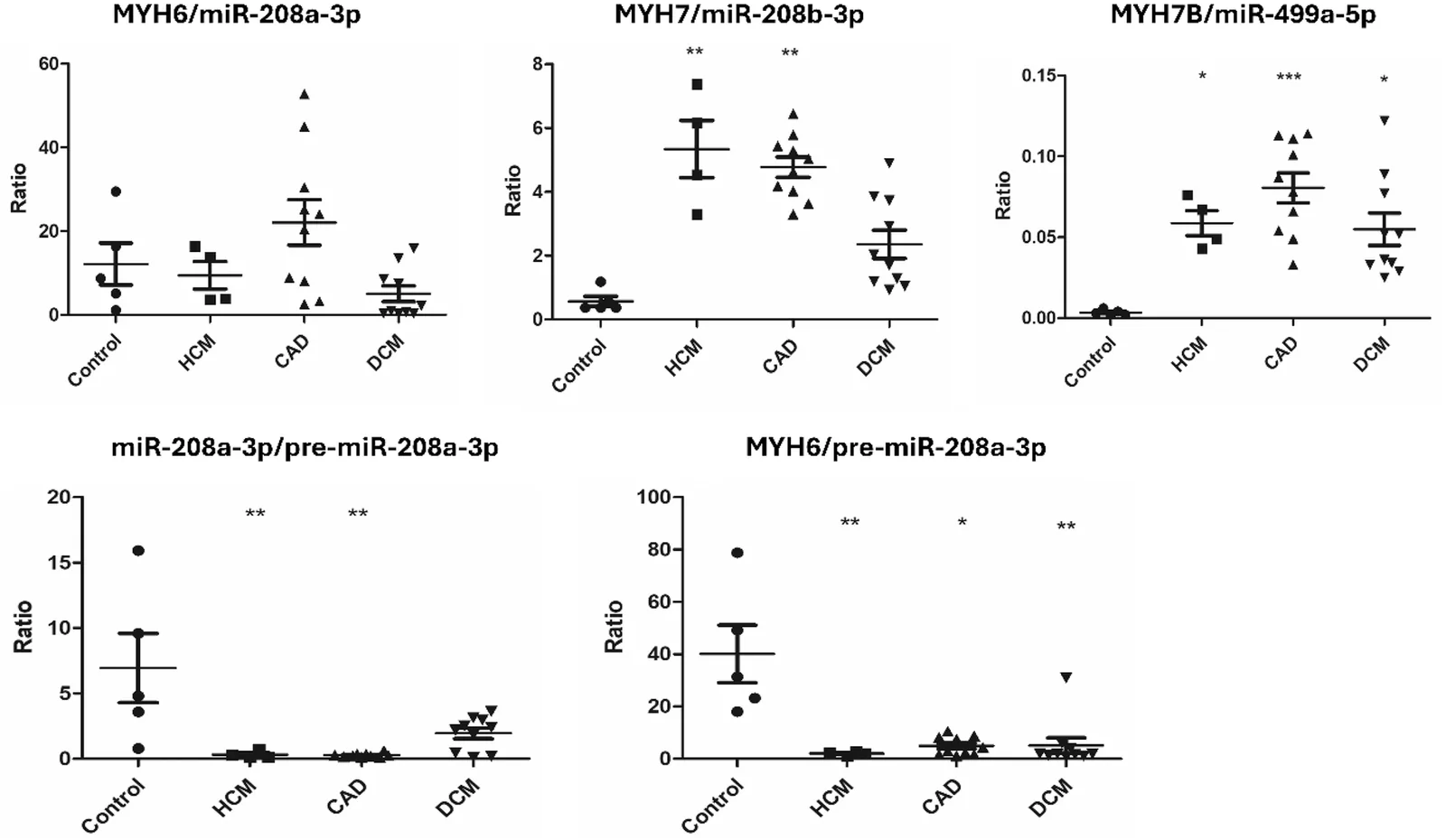
Ratios between myosin transcripts and their corresponding miRNAs/pre-miRNAs. Individual plots show ratios calculated from unnormalised expression data (mean plus/minus SEM). Statistical significance: ***p < 0.001, **p < 0.01, *p < 0.05. HCM, hypertrophic cardiomyopathy; CAD, coronary artery disease; DCM, dilated cardiomyopathy.

In contrast, *miR-208b-3p* expression, which is high in healthy tissue, remained largely unchanged across disease states, underscoring its dominant role among the MYH-derived microRNAs. The *miR-208a-3p/miR-208b-3p* ratio was heavily skewed toward *miR-208b-3p* in all groups. For instance, in control samples, *MYH6* mRNA accounted for approximately 37% of total myosin expression, whereas *miR-208a-3p* represented only ∼3.5% of total miR-208 levels, a decrease further exacerbated in cardiomyopathies (Figure 5; Supplementary SM7 Table 1; Supplementary SM8 Table 1). Despite the coordinated transcription of *MYH7B* and *MYH7* genes, the corresponding miRNA expression ratios (*miR-499a-5p/miR-208b-3p*) varied significantly in diseased tissues, with approximately twofold reductions in CAD and DCM (Figure 5; Supplementary SM7 Table 2; Supplementary SM8 Table 1). The *MYH7/miR-208b-3p* and *MYH7B/miR-499a-5p* ratios were significantly elevated in diseased hearts, reflecting the upregulation of the host transcripts. In contrast, the *MYH6/miR-208a-3p* ratio remained stable, suggesting coordinated regulation (Figure 6; Supplementary SM7 Tables 4–6; Supplementary SM8 Tables 2–4).

This is further supported by consistent expression patterns in *MYH6/pre-miR-208a* and *miR-208a-3p/pre-miR-208a* ratios (Figure 6; Supplementary SM7 Tables 7, 8; Supplementary SM8 Table 5). Notably, the expression of *miR-208b-3p* and *miR-499a-5p* appears to be regulated independently of their host transcripts (Figure 6; Supplementary SM8 Tables 3, 4). Additionally, a pronounced imbalance in the MYH7B/*miR-499a-5p* ratio was observed. In healthy controls, this ratio was approximately 250:1 in favour of the miRNA (Supplementary SM8 Table 4), indicating a possible uncoupling of transcript and miRNA expression. This imbalance increased 5- to 10-fold in diseased tissues, suggesting that despite low transcript levels, the *MYH7B* gene locus may still play a significant functional role via its miRNA product.

### 3.4 The *miR-208a-3p/TARBP2/SOX6* axis in human cardiac hypertrophy

**Figure 7:**
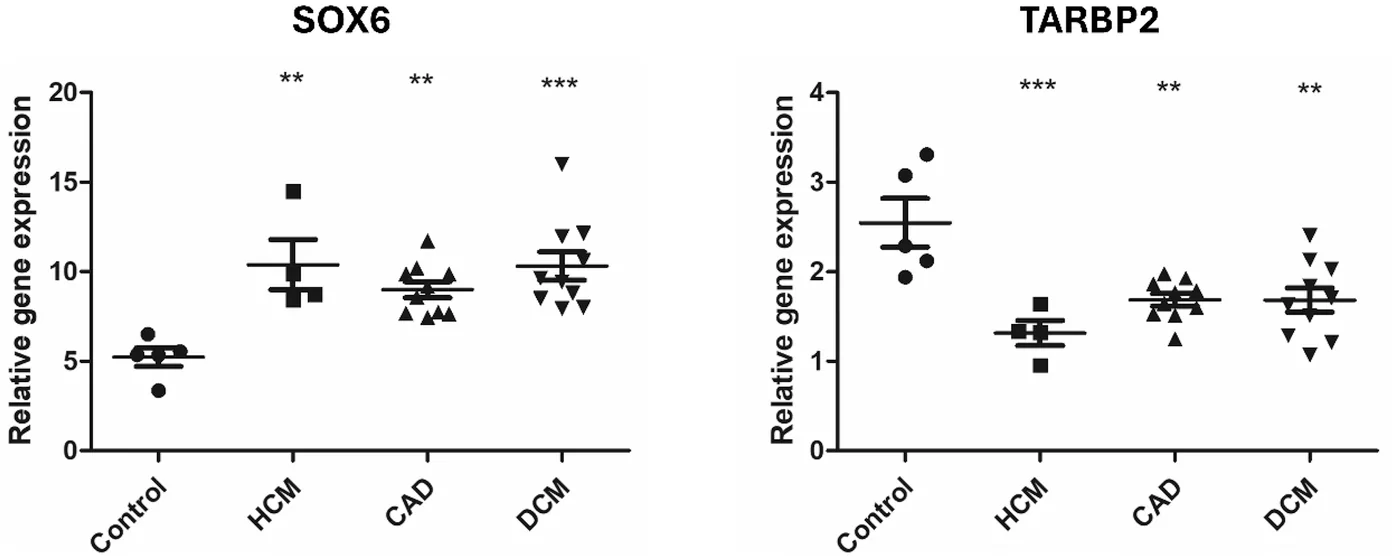
Relative expression of SOX6 and TARBP2 transcripts in non-failing and failing human left ventricles. Plots indicate the mean gene expression plus/minus SEM. Statistical significance is indicated as: ***p < 0.001, **p < 0.01, *p < 0.05. HCM, hypertrophic cardiomyopathy; CAD, coronary artery disease; DCM, dilated cardiomyopathy.

In murine hearts, the *miR-208a-3p/TARBP2/SOX6* regulator*y* axis is a key modulator of contractile protein expression; specifically, *miR-208a-3p* downregulation leads to increased *SOX6* levels, which in turn suppresses *MYH7* and *MYH7B* transcription (18, 34). However, the specific roles of SOX6 and TARBP2 in regulating human myosin heavy chains remain poorly characterised. In our human samples, TARBP2 transcript levels were modestly but significantly decreased across all pathological groups, while SOX6 mRNA expression was significantly upregulated in diseased tissues (Figure 7; Supplementary SM9 Tables 1, 2). These findings suggest that the miR-208a-3p/TARBP2/SOX6 axis is actively involved in the transcriptional response to hypertrophic stimuli in human hearts, showing a conserved upstream activation pattern, even though its downstream genetic outcomes diverge from those in rodents.

## 4 Discussion

In this study, we explored the expression profiles of *MYH6*, *MYH7*, *MYH7B*, and *MHRT* transcripts, alongside their corresponding myomiRs (*miR-208a-3p, miR-208b-3p*, and *miR-499a-5p*), across three distinct cardiomyopathy cohorts. Our results demonstrate that *MYH6* is consistently downregulated, whereas *MYH7* is significantly upregulated across all pathological groups (Figure 1), a classic hallmark of cardiac remodelling (13, 15). A key finding of this analysis is that the *MYH6/MYH7* transcript ratio exhibits substantially greater variation in non-failing control hearts than in the left ventricles of failing hearts, consistent with previously reported findings (13). Given that a higher *MYH6/MYH7* ratio is associated with preserved cardiac function (35), we examined this ratio in specific cardiomyopathies. We found that, across all hypertrophic conditions, it is markedly decreased, regardless of age or gender (Supplementary SM3 Table 1).

Our results further indicate a potential coordination of *MYH7*, *MYH7B*, and *MHRT* transcription in the human left ventricle. Specifically, we observed that *MYH7*, *MHRT*, and *MYH7B* transcripts are consistently upregulated, whereas *MYH6* is downregulated in diseased tissues. In rodents, *MHRT* expression is typically coordinated with *MYH6* levels and is regulated by specific epigenetic factors (20, 22). According to the established murine model, the release of the *Ezh2/pri-miR-208b/HDAC2* complex from the *MHRT* promoter (bdP) leads to a concomitant increase in both *MYH6* and *MHRT*, which in turn suppresses *MYH7* transcription.

However, our findings in the failing human heart are not consistent with this model, as *MYH7* transcript levels were significantly increased alongside *MHRT* expression. Conversely, the relationship between *MYH6* and *MHRT* in our human samples mimics observations in neonatal rat cardiomyocytes, in which *MHRT* upregulation was associated with *MYH6* transcript downregulation (25). Furthermore, the correlation between *MYH7* and *MYH7B* expression in human left ventricles, which we observed in the GSE116250 dataset and in our experimental findings, has been previously reported in human induced pluripotent stem cell-derived cardiomyocytes (hiPSCMs) (27). Taken together, our results suggest that the coordination of these three transcripts differs significantly between rodents and humans. This likely reflects distinct evolutionary adaptations and divergent molecular responses to hypertrophic stimuli, underscoring the limitations of using murine models to fully explain human cardiac pathophysiology.

As mentioned above, concurrent changes in *MYH6* and *MYH7* expression (the *MYH6*/*MYH7* ratio) are hallmarks of cardiac hypertrophy and heart failure. Much less is known, however, about the relationship between their corresponding *miRNAs, miR-208a-3p* and *miR-208b-3p*. Our analysis of the *miR-208a-3p/miR-208b-3p* ratio across both healthy and diseased groups showed that this ratio is strongly shifted in favour of *miR-208b-3p* (Supplementary SM7 Table 1), while *miR-208a-3p* remains at very low basal levels. Interestingly, in the left ventricles of healthy hearts, this miRNA ratio was on average tenfold lower than the *MYH6*/*MYH7* transcript ratio. This discrepancy indicates a significant disconnection between *miR-208a-3p* and its host gene, MYH6, under normal physiological conditions. On the other hand, the *miR-208a-3p*/*miR-208b-3p* values were consistent between healthy and diseased groups and generally followed the downward trend of the *MYH6*/*MYH7* ratios in the pathological cohorts.

Interestingly, these low ratios raise a fundamental question regarding the functional role of *miR-208a-3p* in the human left ventricle. In the human myocardium, both *MYH6* and *MYH7* transcripts are present (with *MYH6* representing ∼36% of total myosin content in non-failing LVs) (13), alongside all three myomiRs derived from the myosin heavy chain (MHC) genes. Since *miR-208a-3p* persists in diseased tissues—albeit at much lower levels than *miR-208b-3p*—it likely maintains an adaptive function. One potential role may be the post-transcriptional regulation of a distinct set of target genes essential for cardiac performance, similar to the known functions of *miR-208b-3p*. Notably, both miRNAs possess an identical seed sequence and differ at only three positions outside of the mRNA binding region. These subtle sequence variations may confer the ability to distinguish between specific targets for *miR-208a-3p* and *miR-208b-3p*, respectively. Furthermore, as *miR-208a-3p* has been shown to regulate *miR-499a-5p* expression in the murine heart (18), it is plausible that a similar regulatory hierarchy exists in the human myocardium.

Noteworthy, the expression pattern of the *miR-208a-3p*/*miR-208b-3p* ratio observed in our datasets is reminiscent of previously reported data on *α*-MHC protein levels in non-failing and failing human left ventricles (13, 14). In those studies, *α*-MHC accounted for only about 2.5% of the total myosin content in healthy LVs, a value that was further reduced by 2- to 8-fold in failing hearts (14). Similarly, our results show that *miR-208a-3p* represents just 3.5% of the total miR-208 pool (*miR-208a-3p* + *miR-208b-3p*) in control samples, with a further 2- to 5-fold decrease observed in the pathological groups (Supplementary SM7 Table 1). This striking correlation between the miRNA and the protein levels of their host genes suggests that the transcriptional and post-transcriptional regulation of the *MYH6* locus is tightly maintained, even if the absolute levels of its products are low in the human heart compared to other species.

The expression analysis of *MYH7*B, another member of the sarcomeric myosin-II family, showed significant upregulation in the left ventricles of diseased myocardium, consistent with previously published findings (36). Evaluation of the *MYH7*B/*MYH7* ratio demonstrated a strong predominance of the *MYH7* transcript, with *MYH7*B accounting for less than 1% of the combined *MYH7* and *MYH7*B transcript pool (Supplementary SM7 Table 4; Supplementary SM8 Table 1). Notably, *MYH7*B exhibited a transcriptional profile remarkably similar to *MYH7*, with both genes being upregulated in diseased hearts. Despite these expressional shifts, the *MYH7*B/*MYH7* ratio remained stable between non-failing and pathological LVs, a finding further supported by our analysis of larger RNA-seq cohorts (Figure 3; Supplementary SM5 Table 2). These observations align with previous *in vitro* studies (27), which showed that knockdown of a *MYH7*B-derived lncRNA reduced *MYH7* expression, suggesting a coordinated regulatory relationship between these two loci. Interestingly, further analysis revealed that *miR-499a-5p* and *miR-208b-3p*—originating from *MYH7*B and *MYH7* pre-mRNAs, respectively—displayed markedly different abundance patterns than their host transcripts, suggesting an additional layer of post-transcriptional control.

The observed increase in *MYH7*B expression, coupled with decreased levels of *miR-499a-5p* in diseased samples, led us to hypothesise that the *MYH7*B transcript might function as a miRNA “sponge.” However, our in silico analysis of both the full-length *MYH7*B transcript and the exon 10-skipped isoform (*MYH7*B-201) did not reveal canonical *miR-499a-5p* binding sites, arguing against a classical sequestering mechanism. Additional exploratory analysis of *MYH7*B/*miR-499a-5p* ratios (Supplementary SM8 Table 4) showed a consistent shift across all cardiomyopathy groups, with an approximately tenfold increase in diseased hearts compared to controls. This finding suggests that *MYH7*B likely serves regulatory functions beyond merely acting as a precursor for *miR-499a-5p* (36, 37). This interpretation is further supported by studies in human induced pluripotent stem cell-derived cardiomyocytes (hiPSCMs), where the knockdown of *MYH7*B-derived lncRNA not only reduced *MYH7* expression but also downregulated genes essential for sarcomere organisation and contractile function (27). Although based on unnormalised data, these observations collectively support a significant decoupling between *MYH7*B transcript levels and *miR-499a-5p* expression in the failing human heart. Our findings reveal a pronounced discrepancy between the transcriptional induction of the MYH7B host gene and the concomitant downregulation of its intronic miRNA, miR-499a-5p, in failing human hearts (Figures 1B, 5). Although miR-499a-5p is nested within the MYH7B locus and co-transcribed as a single primary transcript, the steady-state levels of mature intronic myomiRs can become significantly uncoupled from their host transcripts during advanced cardiac pathology. It has been established that while host myosin heavy chain transcripts can become induced under stress, the levels of mature miR-499 are specifically downregulated in response to severe cellular and ischemic stress within the myocardium. This transcript-miRNA uncoupling indicates that active transcription of the MYH7B locus is preserved during remodelling, but the post-transcriptional maturation efficiency of miR-499a-5p is compromised (38).

The *MYH7*B/*MYH6* ratio was also found to be very low in the control group but was strongly increased in the compromised left ventricles of all cardiomyopathy cohorts. In this context, the miR-499a-5p/*miR-208a-3p* ratio indicated significantly higher levels of *miR-499a-5p* than *miR-208a-3p* in diseased tissues. Interestingly, this miRNA ratio was slightly reduced in two of the pathological groups, exhibiting the opposite trend compared to the corresponding mRNA ratios (Supplementary SM8 Table 1). The long non-coding RNA *MHRT*, transcribed from a bidirectional promoter located between the *MYH6* and *MYH7* genes, is a chromatin remodeling factor critical in cardiac hypertrophy (20, 22). In stressed rodent hearts, the SWI/SNF chromatin remodeling complex subunit Brg1 typically represses *MYH6* transcription and activates the *MYH7* gene, thereby decreasing the *MYH6*/*MYH7* ratio. However, the *MHRT*–Brg1 interaction is known to reduce Brg1 activity at the *MYH6* promoter and repress *MYH7* expression, exerting an anti-hypertrophic effect. While previous studies reported a downregulation of this lncRNA in stressed human LVs (20), we observed the opposite effect: *MHRT* levels were significantly increased in all diseased samples compared to controls. This is consistent with reported *MHRT* upregulation in patients with obstructive HCM (39) and is further supported by our analysis of two independent RNA-seq datasets (Figure 4; Supplementary SM5 Table 3). One possible explanation for these inconsistencies is that *MHRT* expression in compromised hearts may depend on the severity of the disease. In hearts on the verge of collapse, *MHRT* levels may increase as a compensatory mechanism to attenuate the failure of cardiac contractility. This hypothesis is supported by observations that remote ischemic preconditioning, which improves hemodynamic parameters and protects the myocardium in coronary disease patients, is associated with a subsequent reduction in *MHRT* transcript levels (34).

Importantly, the regulation of *MYH6*, *MYH7*, and *MYH7*B expression via myomiR modulators is not a direct process. Instead, myosin transcript levels are controlled through complex signaling pathways involving both miRNAs and specific transcriptional activators or repressors (18, 40). In this context, *miR-208a-3p* is a key component of the *miR-208a-3p*/*TARBP2/SOX6* axis, which has been shown to modulate hypertrophy in murine models (40). Specifically, *TARBP2* selectively regulates the biogenesis of a subset of miRNAs, including *miR-208a-3p* and *miR-499a-5p*; both are required to suppress SOX6 function and maintain the balance of specific myofibers in the heart (40). Furthermore, aldosterone has been reported to inhibit the fetal gene program in transgenic mice via the inhibition of *miR-208a-3p* and subsequent upregulation of *SOX6*, which in turn represses *MYH7* expression (41). However, our observations do not support this specific regulatory model in the human heart. Our analysis of *SOX6* expression in failing human LVs revealed significantly elevated transcript levels across all three disease groups (Figure 7; Supplementary SM9 Table 1), consistent with its known upregulation in patients with HCM (39).

Crucially, in our human samples, elevated *SOX6* expression coincided with the upregulation of *MYH7* and *MYH7*B. This mirrors findings in HCM patients (39) but directly contrasts with the regulatory patterns described in murine hearts, where SOX6 acts as a repressor of these genes (40). This discrepancy likely reflects evolutionary differences in the requirements for specific myosin isoform ratios between rodent and human myocardium. At the same time, *TARBP2* expression was significantly downregulated in diseased human hearts (Figure 7; Supplementary SM9 Table 2). This downregulation may lead to impaired processing of *miR-208a-3p*, allowing for higher *SOX6* expression, which in turn influences the transcriptional remodeling associated with heart failure.

## 5 Conclusions

In summary, our results demonstrate that *MYH7*, *MYH7*B, and *MHRT* transcripts exhibit highly correlated expression patterns in the human left ventricle, independent of the underlying cardiomyopathy or pathological status. We identified a significant dissociation between *miR-208a-3p* and its host gene (*MYH6*) under physiological conditions and confirmed altered miR-499a-5p/*MYH7*B relationships, consistent with previous findings in murine models. Notably, *miR-208a-3p* expression more closely parallels *MYH6* protein levels rather than transcript abundance, suggesting an additional layer of post-transcriptional control. Furthermore, our analysis of the regulatory genes SOX6 and TARBP2 revealed that SOX6 expression is significantly increased across all pathological groups. This may reflect a regulatory pattern in human heart tissue that differs from murine models, where SOX6 upregulation is typically associated with reduced *MYH7* and *MYH7*B expression. Whether SOX6 contributes to these species-specific differences remains to be determined experimentally. To our knowledge, this is also the first study to examine *TARBP2* expression in healthy and diseased human hearts. We observed a consistent reduction of *TARBP2* in diseased tissue, suggesting it may play a critical role in *miR-208a-3p* biogenesis in the human heart, mirroring its proposed function in murine models. Collectively, these findings suggest that the miR-208a-3p/*TARBP2*/*SOX6* axis is a key contributor to the pathological remodeling of myosin isoforms in the compromised human heart.

## 6 Study limitations

There are several limitations to this study that should be acknowledged.

First, the sample size is relatively small, with all cohorts containing a limited number of samples, though these represent highly unique and clinically valuable human specimens. To mitigate this limitation and strengthen the robustness of our findings, we successfully validated our core results using two independent, larger public RNA-seq datasets.

Second, the non-failing control group consisted of samples from younger donors compared with the diseased groups, which could introduce age-related confounding factors. In addition, clinical heterogeneity among patients—including variations in disease duration, various non-clusterable comorbidities, and individualized pharmacological regimens—represents potential confounders. However, since all individuals in the pathological cohorts were strictly end-stage heart failure patients requiring transplantation, their clinical management followed highly standardized, guideline-directed medical therapies, minimizing the impact of treatment variation on the dominant transcriptomic shifts.

Third, RT-qPCR analysis was performed on cardiac tissue. As the myocardium is a complex tissue containing not only cardiomyocytes but also fibroblasts, endothelial cells, and immune cells, the observed changes—particularly in SOX6 and TARBP2 expression—may not be exclusively myocyte-specific.

Fourth, *ex vivo* investigation provides only a temporal snapshot of the transcriptional status at the time of tissue collection. Consequently, this cross-sectional design identifies robust associations but does not establish direct causal relationships across different stages of disease progression.

Fifth, this study focuses primarily on transcript-level analyses. Although the abundance of miR-208a-3p closely matches established historical data on human alpha-MHC protein levels (13, 14), the lack of matched protein expression analysis remains a limitation. Finally, due to the limited availability of human samples, the gender ratio between the control and pathological groups was not perfectly balanced.

## Data Availability

The datasets presented in this study can be found in online repositories. The names of the repository/repositories and accession number(s) can be found in the article/Supplementary Material.
