# Supplementary material for: Integrated analysis of MYH6, MYH7, and MYH7B expression and their associated microRNAs in human end-stage heart failure
Source: Front Cardiovasc Med. 2026 Aug 7;13:1879458. doi: 10.3389/fcvm.2026.1879458 (PMC13493238; doi:10.3389/fcvm.2026.1879458)
Supplement: Supplementary file 1 [file Supplementaryfile2.docx]

**Statistical analysis of relative gene expression of *MYH6, MYH7, MYH7B* and *MHRT* transcripts in controls and three different cardiomyopathies**

Table 1.

*MYH6*

|  | Control | HCM | CAD | DCM |
| --- | --- | --- | --- | --- |
| Control | 1 | **0.003** | **0.017** | **0.0004** |
| HCM |  | 1 | 0.258 | 0.937 |
| CAD |  |  | 1 | 0.164 |
| DCM |  |  |  | 1 |

Kruskal-Wallis test, *p* **= 0.0004**

Table 2.

*MYH7*

|  | Control | HCM | CAD | DCM |
| --- | --- | --- | --- | --- |
| Control | 1 | **0.040** | **<0.0001** | **0.015** |
| HCM |  | 1 | 0.162 | 0.929 |
| CAD |  |  | 1 | **0.049** |
| DCM |  |  |  | 1 |

Kruskal–Wallis test, *p* **< 0.0001**

Table 3.

*MYH7B*

|  | Control | HCM | CAD | DCM |
| --- | --- | --- | --- | --- |
| Control | 1 | 0.073 | **0.001** | **0.001** |
| HCM |  | 1 | 0.346 | 0.279 |
| CAD |  |  | 1 | 0.854 |
| DCM |  |  |  | 1 |

Kruskal–Wallis test, *p* = **0.0008**

Table 4.

*MHRT*

|  | Control | HCM | CAD | DCM |
| --- | --- | --- | --- | --- |
| Control | 1 | **0.003** | **<0.0001** | 0.056 |
| HCM |  | 1 | 0.627 | 0.119 |
| CAD |  |  | 1 | **0.007** |
| DCM |  |  |  | 1 |

Kruskal–Wallis test, *p* < **0.0001**

Statistical analysis was performed using the Kruskal–Wallis test, followed by Dunn’s post hoc test for multiple comparisons. A *p*-value of ≤ 0.05 was considered statistically significant.
