# Supplementary material for: Integrated analysis of MYH6, MYH7, and MYH7B expression and their associated microRNAs in human end-stage heart failure
Source: Front Cardiovasc Med. 2026 Aug 7;13:1879458. doi: 10.3389/fcvm.2026.1879458 (PMC13493238; doi:10.3389/fcvm.2026.1879458)
Supplement: Supplementary file 2 [file Supplementaryfile4.docx]

**Statistical analysis of the all *MYH6, MYH7. MYH7B* and *MHRT* transcripts ratios in controls and three different cardiomyopathies**

Table 1.

*MYH6/MYH7*

|  | Control | HCM | CAD | DCM |
| --- | --- | --- | --- | --- |
| Control | 1 | **0.007** | **0.007** | **0.001** |
| HCM |  | 1 | 0.565 | 0.921 |
| CAD |  |  | 1 | 0.372 |
| DCM |  |  |  | 1 |

Kruskal–Wallis test, *p* = **0.0019**

Table 2.

*MYH6/MYH7B*

|  | Control | HCM | CAD | DCM |
| --- | --- | --- | --- | --- |
| Control | 1 | **0.003** | **0.019** | **0.0002** |
| HCM |  | 1 | 0.246 | 0.929 |
| CAD |  |  | 1 | 0.098 |
| DCM |  |  |  | 1 |

Kruskal–Wallis test, *p* = **0.0005**

Table 3.

*MYH6/MHRT*

|  | Control | HCM | CAD | DCM |
| --- | --- | --- | --- | --- |
| Control | 1 | **0.002** | **0.005** | **0.002** |
| HCM |  | 1 | 0.321 | 0.500 |
| CAD |  |  | 1 | 0.674 |
| DCM |  |  |  | 1 |

Kruskal–Wallis test, *p* = **0.001**

Table 4.

*MYH7B/MYH7*

|  | Control | HCM | CAD | DCM |
| --- | --- | --- | --- | --- |
| Control | 1 | 0.951 | 0.732 | 0.191 |
| HCM |  | 1 | 0.699 | 0.254 |
| CAD |  |  | 1 | 0.043* |
| DCM |  |  |  | 1 |

Kruskal–Wallis test, *p* = 0.214

Table 5.

*MYH7B*/*MHRT*

|  | Control | HCM | CAD | DCM |
| --- | --- | --- | --- | --- |
| Control | 1 | 0.806 | 0.966 | **0.016** |
| HCM |  | 1 | 0.812 | **0.012** |
| CAD |  |  | 1 | **0.003** |
| DCM |  |  |  | 1 |

Kruskal–Wallis test, *p* = **0.0022**

Table 6.

*MYH7*/*MHRT*

|  | Control | HCM | CAD | DCM |
| --- | --- | --- | --- | --- |
| Control | 1 | 0.259 | 0.534 | 0.668 |
| HCM |  | 1 | 0.481 | 0.093 |
| CAD |  |  | 1 | 0.198 |
| DCM |  |  |  | 1 |

Kruskal–Wallis test, *p* = 0.346

Statistical analysis was performed using the Kruskal–Wallis test, followed by Dunn’s post hoc test for multiple comparisons. A p-value of ≤ 0.05 was considered statistically significant.

*Although this pairwise comparison reached nominal significance (p < 0.05), the overall Kruskal–Wallis test was not significant; therefore, these differences were not considered statistically robust.
