# Supplementary material for: Integrated analysis of MYH6, MYH7, and MYH7B expression and their associated microRNAs in human end-stage heart failure
Source: Front Cardiovasc Med. 2026 Aug 7;13:1879458. doi: 10.3389/fcvm.2026.1879458 (PMC13493238; doi:10.3389/fcvm.2026.1879458)
Supplement: Supplementary file 3 [file Supplementaryfile6.docx]

**Statistical analysis of the relative gene expression of miRNA-208a-3p, miRNA-208b-3p and miRNA-499a-5p transcripts in controls and three different cardiomyopathies**

Table 1.

miRNA-208a-3p

|  | Control | HCM | CAD | DCM |
| --- | --- | --- | --- | --- |
| Control | 1 | **0.019** | **0.002** | 0.592 |
| HCM |  | 1 | 0.866 | **0.031** |
| CAD |  |  | 1 | **0.002** |
| DCM |  |  |  | 1 |

Kruskal–Wallis test, *p* **= 0.0001**

Table 2.

miRNA-208b-3p

|  | Control | HCM | CAD | DCM |
| --- | --- | --- | --- | --- |
| Control | 1 | 0.078 | 0.748 | 0.191 |
| HCM |  | 1 | 0.09 | **0.001** |
| CAD |  |  | 1 | **0.046** |
| DCM |  |  |  | 1 |

Kruskal–Wallis test, *p* = **0.006**

Table 3.

miRNA-499a-5p

|  | Control | HCM | CAD | DCM |
| --- | --- | --- | --- | --- |
| Control | 1 | **0.014** | **0.001** | 0.094 |
| HCM |  | 1 | 0.751 | 0.218 |
| CAD |  |  | 1 | **0.041** |
| DCM |  |  |  | 1 |

Kruskal–Wallis test, *p* = **0.002**

Statistical analysis was performed using the Kruskal–Wallis test, followed by Dunn’s post hoc test for multiple comparisons. A *p*-value of ≤ 0.05 was considered statistically significant.
