# Supplementary material for: Integrated analysis of MYH6, MYH7, and MYH7B expression and their associated microRNAs in human end-stage heart failure
Source: Front Cardiovasc Med. 2026 Aug 7;13:1879458. doi: 10.3389/fcvm.2026.1879458 (PMC13493238; doi:10.3389/fcvm.2026.1879458)
Supplement: Supplementary file 4 [file Supplementaryfile7.docx]

**Statistical analysis of the relative gene expression of miRNA-208a-3p/miRNA-208b-3p, miRNA-499a-5p/miRNA-208b-3p and miRNA 208a-3p/miRNA-499a-5p transcript ratios in controls and three different cardiomyopathies**

Table 1

**miRNA-208a-3p/miRNA-208b-3p**

|  | Control | HCM | CAD | DCM |
| --- | --- | --- | --- | --- |
| Control | 1 | **0.023** | **<0.0001** | 0.079 |
| HCM |  | 1 | 0,275 | 0.341 |
| CAD |  |  | 1 | **0.007** |
| DCM |  |  |  | 1 |

Kruskal–Wallis test, *p* **= 0.0001**

Table 2

**miRNA-499a-5p/miRNA-208b-3p**

|  | **Control** | **HCM** | **CAD** | **DCM** |
| --- | --- | --- | --- | --- |
| **Control** | 1 | 0.323 | **0.002** | **0.001** |
| **HCM** |  | 1 | 0.076 | **0.036** |
| **CAD** |  |  | 1 | 0.674 |
| **DCM** |  |  |  | 1 |

Kruskal–Wallis test, *p* **= 0.0004**

Table 3

**miRNA-208a-3p/miRNA-499a-5p**

|  | **Control** | **HCM** | **CAD** | **DCM** |
| --- | --- | --- | --- | --- |
| **Control** | 1 | 0.070 | **0.036** | 0.898 |
| **HCM** |  | 1 | 0.913 | **0.030** |
| **CAD** |  |  | 1 | **0.006** |
| **DCM** |  |  |  | 1 |

Kruskal–Wallis test, *p* **= 0.006**

**Statistical analysis of the relative gene expression of *MYH6*/ miRNA-208a-3p, *MYH7*/miRNA-208b-3p, *MYH7B*/miRNA-499a-5p transcript ratios in controls and three different cardiomyopathies**

Table 4

***MYH6*/ miRNA-208a-3p**

|  | Control | HCM | CAD | DCM |
| --- | --- | --- | --- | --- |
| Control | 1 | 0.840 | 0.391 | 0.103 |
| HCM |  | 1 | 0.307 | 0.200 |
| CAD |  |  | 1 | **0.002** |
| DCM |  |  |  | 1 |

Kruskal–Wallis test, *p* = **0.015**

Table 5

***MYH7*/miRNA-208b-3p**

|  | **Control** | **HCM** | **CAD** | **DCM** |
| --- | --- | --- | --- | --- |
| **Control** | 1 | **0.001** | **0.0001** | 0.079 |
| **HCM** |  | 1 | 0.850 | **0.034** |
| **CAD** |  |  | 1 | **0.011** |
| **DCM** |  |  |  | 1 |

Kruskal–Wallis test, *p* **<0.0001**

Table 6

***MYH7B*/miRNA-499a-5p**

|  | **Control** | **HCM** | **CAD** | **DCM** |
| --- | --- | --- | --- | --- |
| **Control** | 1 | **0.023** | **0.0001** | **0.011** |
| **HCM** |  | 1 | 0.341 | 0.812 |
| **CAD** |  |  | 1 | 0.115 |
| **DCM** |  |  |  | 1 |

Kruskal–Wallis test, *p* **= 0.0003**

**Statistical analysis of the relative gene expression of *MYH6*/pre-miRNA-208 and miRNA-208a-3p/pre-miRNA-208 transcript ratios in control and three different cardiomyopathies**

Table 7

***MYH6*/pre-miRNA-208**

|  | **Control** | **HCM** | **CAD** | **DCM** |
| --- | --- | --- | --- | --- |
| **Control** | 1 | **0.006** | **0.015** | **0.001** |
| **HCM** |  | 1 | 0.366 | 0.976 |
| **CAD** |  |  | 1 | 0.217 |
| **DCM** |  |  |  | 1 |

Kruskal–Wallis test, *p* = **0.001**

Table 8

**miRNA-208a-3p/pre-miRNA-208**

|  | **Control** | **HCM** | **CAD** | **DCM** |
| --- | --- | --- | --- | --- |
| **Control** | 1 | **0.004** | **0.001** | 0.108 |
| **HCM** |  | 1 | 0.953 | 0.077 |
| **CAD** |  |  | 1 | **0.024** |
| **DCM** |  |  |  | 1 |

Kruskal–Wallis test, *p* = **0.0004**

Statistical analysis was performed using the Kruskal–Wallis test, followed by Dunn’s post hoc test for multiple comparisons. A *p*-value of ≤ 0.05 was considered statistically significant.
