# Supplementary material for: Integrated analysis of MYH6, MYH7, and MYH7B expression and their associated microRNAs in human end-stage heart failure
Source: Front Cardiovasc Med. 2026 Aug 7;13:1879458. doi: 10.3389/fcvm.2026.1879458 (PMC13493238; doi:10.3389/fcvm.2026.1879458)
Supplement: Supplementary file 5 [file Supplementaryfile9.docx]

**Statistical analysis of the mRNA expression of *SOX6* and *TARBP2* genes in three different cardiomyopathies**

Table 1.

***SOX6***

|  | **Control** | **HCM** | **CAD** | **DCM** |
| --- | --- | --- | --- | --- |
| **Control** | 1 | **0.005** | **0.009** | **0.0005** |
| **HCM** |  | 1 | 0.439 | 0.953 |
| **CAD** |  |  | 1 | 0.270 |
| **DCM** |  |  |  | 1 |

Kruskal–Wallis test, *p* = **0.0013**

Table 2.

***TARBP2***

|  | **Control** | **HCM** | **CAD** | **DCM** |
| --- | --- | --- | --- | --- |
| **Control** | 1 | **0.001** | **0.015** | **0.011** |
| **HCM** |  | 1 | 0.139 | 0.162 |
| **CAD** |  |  | 1 | 0.916 |
| **DCM** |  |  |  | 1 |

Kruskal–Wallis test, *p* = **0.003**

Statistical analysis was performed using the Kruskal–Wallis test, followed by Dunn’s post hoc test for multiple comparisons. Differences were considered statistically significant at p ≤ 0.05.
